# Single‐molecule DNA flow‐stretch assays for high‐throughput DNA–protein interaction studies

**DOI:** 10.1002/2211-5463.70211

**Published:** 2026-02-19

**Authors:** Ayush Kumar Ganguli, Mohammad Nour Alsamsam, Ugnė Bagdonaitė, Van Truc Vu, Chun‐Jen Huang, Polina Kuzhir, Mindaugas Zaremba, Aurimas Kopūstas, Marijonas Tutkus

**Affiliations:** ^1^ Institute of Biotechnology, Life Sciences Center Vilnius University Lithuania; ^2^ Department of Molecular Compound Physics, Center for Physical Sciences and Technology Vilnius Lithuania; ^3^ Department of Chemical & Materials Engineering National Central University Taoyuan Taiwan; ^4^ R&D Center for Membrane Technology Chung Yuan Christian University Chung‐Li City Taiwan; ^5^ School of Materials Science and Engineering The University of New South Wales Sydney Australia; ^6^ Department of Physics and Mathematics, Center for Photonics Sciences University of Eastern Finland Joensuu Finland

**Keywords:** DNA, DNA flow‐stretch, DNA‐protein interactions, flowcell, fluorescence microscopy, single‐molecule biophysics

## Abstract

DNA‐interacting proteins are involved in various molecular processes that are fundamental to cells' health and function. These include essential processes such as replication, DNA damage repair and transcriptional regulation. Additionally, DNA‐interacting proteins have significant application potential in biotechnology, diagnostics and medicine. Single‐molecule techniques enable us to reveal and characterise the behaviour of these proteins, which is typically obscured in molecular biology and biochemistry measurements due to ensemble averaging. Typical low‐complexity single‐molecule assays work great for mechanistic interaction studies. However, they are limited in terms of DNA substrate length and their arrangement. Therefore, the single‐molecule DNA flow‐stretch assays were developed, offering a higher level of complexity and a more natural‐like approach for probing real‐time DNA–protein interactions. They use long linear surface‐tethered DNA fragments that can be stretched along the surface using a buffer flow. Here, we present an optimised protocol that focusses on key steps of the experiment, including glass surface preparation, tethering chemistries and fluorescent labelling and imaging of DNA and proteins. In our protocol, bacteriophage λ DNA provides robust flow‐induced extension, which is particularly suitable for studying proteins that influence the length of the DNA molecule or translocate along it. The shorter phiX DNA is mostly suitable for testing, optimisation and validation of the assay. This protocol outlines critical considerations for enhancing the reproducibility and accessibility of single‐molecule DNA flow‐stretch assays, thereby advancing their application in mechanistic, high‐throughput and higher‐complexity studies of DNA–protein interactions that are widespread across diverse biological systems.

## Graphical abstract

We describe an optimised single‐molecule DNA flow‐stretch assay that visualises DNA–protein interactions in real time. Linear DNA fragments are tethered to a surface and stretched by buffer flow for fluorescence imaging. Using λ and φX174 DNA, this protocol enhances reproducibility and accessibility, providing a versatile approach for studying diverse proteins.

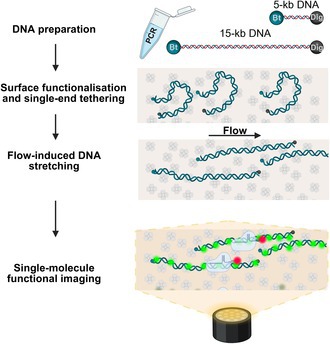

**Abbreviations**
BSA: bovine serum albumine
CRISPR‐Cas9: clustered regularly interspaced short palindromic repeats‐associated protein 9
crRNA: CRISPR RNA
DEPC: diethylpyrocarbonate
DNA: deoxyribonucleic acid
DTT: dithiothreitol
EDTA: ethylenediaminetetraacetic acid
EtOH: ethanol
FOV: field‐of‐view
FRET: Förster resonance energy transfer
FRET: Förster resonance energy transfer
gRNA: guide RNA
kb: kilobase
PBS: Phosphate‐buffered saline
PCR: polymerase chain reaction
PEG: polyethylene glycol
phiX DNA: linear fragment produced from φX174 plasmid DNA
RNP: ribonucleoprotein
RT: room temperature
TIRF: total internal reflection fluorescence
tracrRNA: transactivating CRISPR RNA; →, injection of solution into the flowcell
λ DNA: bacteriophage λ DNA

DNA‐interacting proteins are involved in various processes that are fundamental to the proper functioning of a living cell [1, 2, 3, 4, 5]. Additionally, these proteins have significant application potential in biotechnology, diagnostics and medicine [5, 6, 7, 8, 9, 10, 11, 12, 13]. For instance, CRISPR‐Cas technology has revolutionised the field of genome editing for treating the causes of genetic diseases [14, 15, 16, 17, 18]. Traditional molecular biology and biochemistry bulk methods, used for characterisation of DNA‐interacting proteins, are susceptible to ensemble averaging, which typically obscures the behaviour of single molecules and reports an average value of a given population (trillions of molecules observed altogether). To extract kinetic information in these experiments, a special workaround must be used to synchronise all molecules within the population to the same state at the beginning of the measurement [19].

The single‐molecule techniques, such as tethered particle motion, magnetic or optical tweezers, and FRET, have transformed molecular biology research and understanding of biomolecule behaviour [20, 21, 22, 23, 24, 25, 26, 27]. They enabled direct real‐time observation of biomolecular interactions at the unprecedented spatiotemporal resolution, typically using low‐complexity DNA substrates [25, 26, 27, 28, 29, 30]. The single‐molecule DNA flow‐stretch assays represent a class of methods that mechanically extend individual surface‐tethered long DNA molecules using hydrodynamic shear forces within flowcell to study DNA‐interacting proteins (Fig. 1) [28, 31, 32]. Several approaches have been developed to increase the throughput of the standard DNA flow‐stretch assay, including the DNA Curtains platform [33, 34], DNA skybridge [35], DNA garden [36] and Soft DNA Curtains [32, 37, 38, 39], as well as suspended DNA Curtains [40]. Here, the interplay between fluorescently labelled DNA‐interacting proteins [28, 34, 39, 41, 42, 43, 44, 45, 46] and surface‐tethered DNA, stained with intercalating dyes, is observed using TIRF microscopy. Optionally, double‐tethering of DNA ensures an extended conformation of DNA without flow, allowing for studying interactions under equilibrium conditions. Next, fully or semi‐automated data analysis routines are used to measure DNA length, detect fluorescent spots in microscopy images and extract binding positions of DNA‐interacting proteins more accurately. Also, they allow for extracting characteristic dwell times of DNA‐interacting protein on DNA, or tracking their translocation/sliding along DNA.

**Fig. 1:**
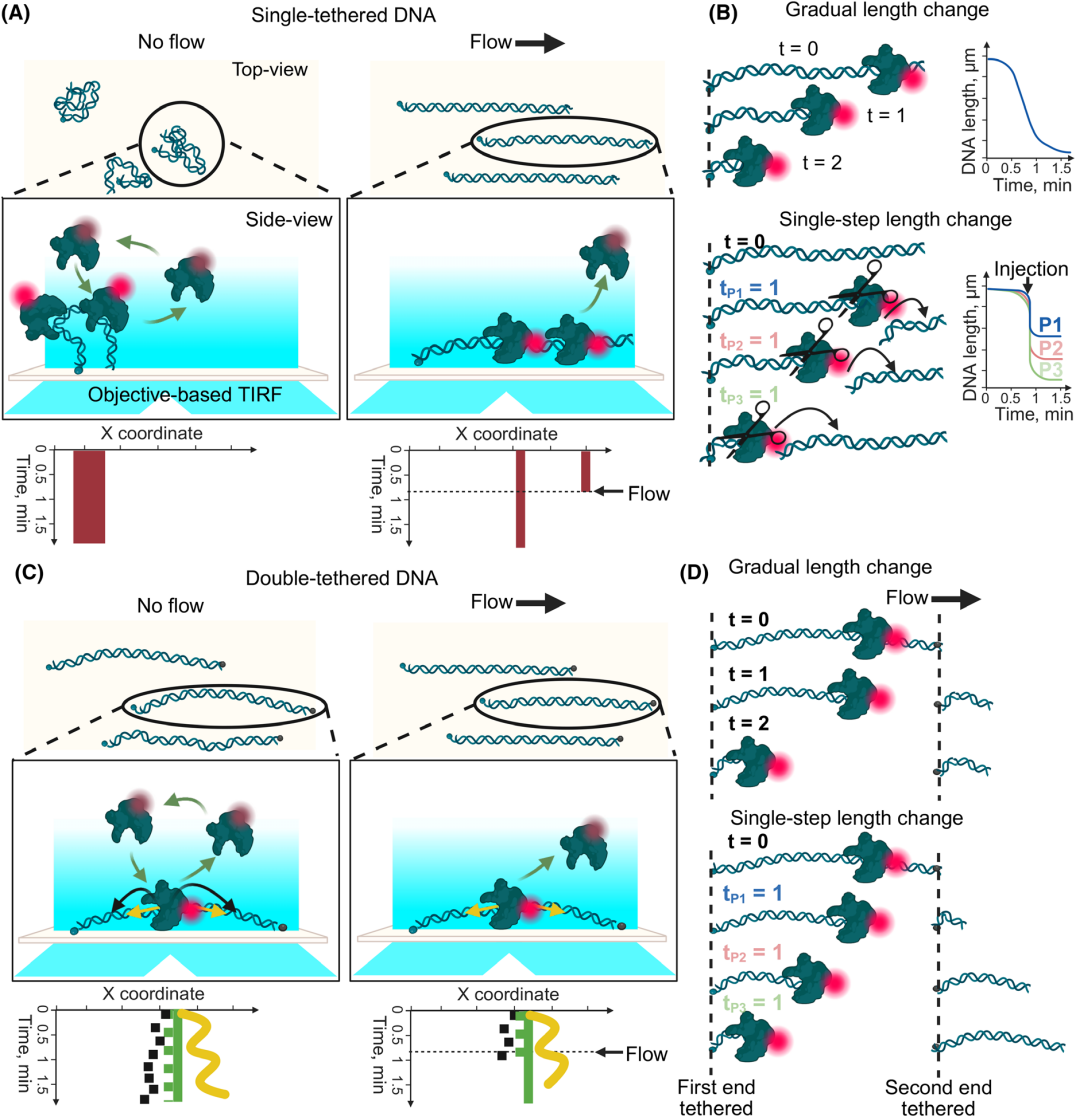
(A) The typical single‐tethered DNA flow‐stretch assay is based on surface tethering of linear DNA fragments and stretching them along the surface using the shear force of the buffer flow in the flowcell. The λ DNA is the substrate of choice when maximal extended DNA length is required. (B) This assay allows for monitoring of DNA cleavage/condensation initial position and dynamics [76]. In this assay, without buffer flow (left panel), DNA‐interacting proteins bind and unbind reversibly (weak interaction) or remain bound for a prolonged period (strong interaction). However, upon initiating the buffer flow (right panel), only the strongly interacting proteins remain bound to the surface‐immobilised DNA fragments. The DNA fragments from 5 to 10 kb length can also be applied for DNA flow‐stretch assays [38]. They allow for optimising the assay and DNA‐interacting protein binding studies that reveal kinetics of binding, but not the position on DNA or sliding/movement. These shorter substrates can also be applied to visualise cleavage of the tethered DNA, but only one cleavage site can be revealed (no gradual shortening). (C) Double tethering allows for maintaining DNA fragments stretched without the need for a buffer flow. This enables the monitoring of binding/unbinding (green‐coloured arrows), sliding (yellow‐coloured arrows), hopping (black‐coloured arrows), and other events of protein interaction with DNA at equilibrium conditions. (D) For the double‐tethered DNA assay, it is possible to perform DNA cleavage position and dynamics of cleavage studies. However, it does not allow for studying DNA‐condensing or ‐looping proteins because both ends of DNA are immobilised. For protein binding position on DNA, sliding/movement studies DNA‐interacting protein has to be labelled fluorescently. *Created in BioRender*. Kopūstas, A. (2025), https://BioRender.com/9gelnhb.

The single‐molecule DNA flow‐stretch assays elucidated mechanisms of a diverse set of DNA‐interacting proteins, demonstrating their broad applicability [31, 37, 41, 47]. However, successfully establishing DNA flow‐stretch assays for routine use in one's laboratory demands careful optimisation of multiple parameters including: DNA substrate preparation, selecting surface chemistry, DNA tethering strategies, choosing fluorescent dyes and troubleshooting. Here, we present a protocol that encompasses detailed guidelines and recommendations and aims to facilitate the broad adoption of the DNA flow‐stretch assay for studying DNA‐interacting proteins at the single‐molecule level.

## Materials

### Reagents

- λ DNA (#SD0011, Thermo Fisher Scientific, Vilnius, Lithuania). Aliquot into smaller volumes and store at −20 °C.
- φX174 DNA plasmid (#SD0031, Thermo Fisher Scientific). Stored at −20 °C.
- Traptavidin—homemade [39].
- Biotinylated antidigoxin antibody (#B7405‐.2ML, Sigma‐Aldrich, Darmstadt, Germany).
- N‐Hydroxysuccinimide‐PEG_4_‐biotin (#2893, Lumiprobe, Hannover, Germany), N‐Hydroxysuccinimide‐PEG_4_‐methyl (#22342, Thermo Fisher Scientific).
- Aminopropyl‐silatrane in ultrapure water (460 mm) and sulfobetaine‐silatrane powder ‐ home‐made [48, 49, 50, 51, 52]. Store at +4 °C.
- Absolute ethanol (#32221, Honeywell, Morris Plains, NJ, USA).
- DMSO (#D12345, Invitrogen, Vilnius, Lithuania).
- Acetic acid (#695092, Sigma‐Aldrich).
- 10X PBS (#70011044, Gibco, Vilnius, Lithuania). Store at room temperature. Dilute to 1X using ultrapure water on the day of the experiment.
- Buffer1 home‐made. Prepare a 10X concentrated solution (330 mm Tris–HCl, 660 mm KCl, pH 7.9 at 23 °C). Filter the solution using 0.22 μm syringe filters, aliquot into smaller volumes, and store at −20 °C. Dilute to 1X using ultrapure water on the day of the experiment.
- SYTOX Green Nucleic Acid Stain (#S7020, Invitrogen). Dilute to 5 μm using DMSO, aliquot in 10 μL, and store at −20 °C.
- Precision glass coverslips (#0107242, Paul Marienfeld, Lauda‐Königshofen, Germany; #1.5, 24 × 60 mm^2^).
- 2‐Propanol (Isopropanol) (#CP41.8, Carl Roth, Karlsruhe, Germany).
- Alcojet (#1401‐1, ALCONOX), prepare a 10 g/L solution using ultrapure water.
- Sodium Hydroxide (#9356.1, Carl Roth), prepare 1 m solution using ultrapure water.
- Ultrapure water (#SYNSVHF00, Merck Millipore, Darmstadt, Germany).
- Double‐sided sticky tape (3 M‐9088‐200, 3 m) with laser‐cut holes and dimensions matching flowcell channels (laser‐cut by “Spaudos idėjos” Vilnius).
- DNA oligonucleotides complementary to λ DNA cosR and cosL sites (ol2: 5′‐GGGCGGCGACCT‐3′ and ol1: 5′‐AGGTCGCCGCCC‐3′, Metabion).
- Biotin‐DNA oligonucleotide complementary to λ DNA cosR site (ol2‐biotin: 5′‐GGGCGGCGACCT‐biotin‐3′, Metabion).
- Digoxigenin‐DNA oligonucleotide complementary to λ DNA cosL site (ol1‐digoxigenin: 5′‐AGGTCGCCGCCC‐C9‐digoxigenin‐3′, Metabion).
- Forward biotin‐DNA oligonucleotide for phiX DNA (ol3‐biotin: 5′‐biotin‐CGAAGTGGACTGCTGGCGG‐3′, Metabion).
- Forward nonmodified DNA oligonucleotide for phiX DNA (ol3: 5′‐CGAAGTGGACTGCTGGCGG‐3′, Metabion).
- Reverse non‐modified DNA oligonucleotide for phiX DNA (ol4: 5′‐CGTAAACAAGCAGTAGTAATTCCTGCTTTATCAAG‐3′, Metabion).
- Bsp120I restriction endonuclease (#ER0131, Thermo Fisher Scientific).
- Biotin (#B4501, Sigma‐Aldrich).
- dNTP Mix (#R1121, Thermo Fisher Scientific).
- Taq DNA Polymerase (#EPO402, Thermo Fisher Scientific).
- Taq Buffer (#B33, Thermo Fisher Scientific).
- Q5 High‐Fidelity DNA Polymerase (#M0491L, #M0491S, New England Biolabs, Frankfurt, Germany).
- Q5 High GC Enhancer (#B9028A, New England Biolabs).
- Q5 Reaction Buffer (#B9027S, New England Biolabs).
- GeneRuler DNA Ladder Mix (#SM0331, Thermo Fisher Scientific).
- 6X MassRuler DNA Loading Dye (#R0621, Thermo Fisher Scientific).
- Catalytically inactive dCas9 from *Streptococcus pyogenes* (in‐house production according to [53]).
- crRNA for dCas9 (5′‐gaaaUccacUgaaagcacag‐3′, Synthego).
- tracrRNA for dCas9 (5′‐GGGCAAAACAGCATAGCAAGTTAAAATAAGGctagtccgttatcaacttgaaaaagtGGCACCGAGTCGGTGCTTTTTGCTCGTGCGC‐3′, in‐house production according to [37]).
- ATTO647N‐labelled DNA oligo (5′‐C6 Amino‐TTGCGCACGAGCAAA‐3′, Metabion; in‐house fluorescently labelled according to [37]).
- 5X annealing buffer (Synthego).
- DEPC‐treated water (#R0601, Thermo Fisher Scientific).
- 10X assembly buffer (in‐house production: 100 mm Tris–HCl (pH 7.5 at 23 °C), 1 M NaCl, 10 mm EDTA, 10 mm DTT).
- 10X reaction buffer (in‐house production: 100 mm Tris–HCl (pH 7.5 at 23 °C), 1 M NaCl, 100 mm MgCl2, 10 mm DTT).
- DTT (#R0861, Thermo Fisher Scientific).

### Equipment

- Peristaltic pump (Ismatec Reglo Digital 2‐channel 8 roller, Darwin MICROFLUIDICS).
- Tubing for peristaltic pump (3‐stop Tygon E‐LFL 0.64 mm ID, Darwin MICROFLUIDICS).
- Adapter silicone tubing to be inserted into the inlet and outlet ports of the flowcell (#CH24.1, Carl Roth).
- Spacer tubing (#Z609706, Bohlender).
- Air plasma cleaner (Diener Zepto W6).
- Objective‐based TIRF microscope, with a sensitive camera and at least two laser lines: 488/638 m or 488/561 nm wavelengths.
- PCR machine (#G8800‐001, Agilent Technologies, Santa Clara, CA, USA).
- Agarose gel electrophoresis (#257286, ClearLine).
- Power Supply (#12643546, Fisherbrand).
- Agarose powder (#R0492, Thermo Fisher Scientific).
- Ethidium bromide (#E1510, Sigma‐Aldrich).
- Gel extraction kit (#K0692, Thermo Fisher Scientific).
- Flowcell (#80608, Ibidi).
- Staining jar (#4200000, Paul Marienfeld).
- Tweezers (#2A.SA.0, Ideal‐Tek).
- Oven (#UN160, memmert).
- Nitrogen gas (Nitrogen 5.0, ELME MESSER GAAS).
- Black permanent marker (#140 S, from edding).
- A single‐use plastic 100 mL container (#25031, FL MEDICAL).
- Tabletop centrifuge with vortexing function (e.g. #FV‐2400, bioSan).
- 50 mL Falcon tubes (#02‐572‐8001, nerbe plus).
- A pH meter for buffer pH adjustment (e.g. #PH700, APERA).
- Centrifugal concentrator (#UFC510024, Merck).

## Methods

### DNA preparation

**Fig. 2:**
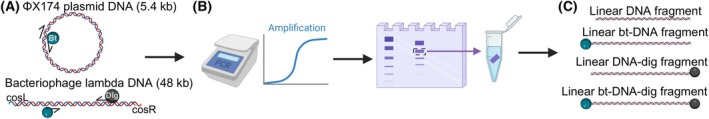
Summary of linear DNA fragment production described in this protocol. (A) Illustrates two substrates (template) and placement of functionalised (digoxigenin (dig), biotin (bt)) or nonfunctionalised DNA oligonucleotide primers for PCR. (B) PCR with these substrates and primers produces desired linear fragments in an amplified fashion. However, unwanted products, primers, and other molecules are still in the PCR mixture. To isolate the DNA fragment of interest, agarose gel electrophoresis is followed by gel cutting and extraction. (C) Illustration of various linear DNA fragments of interest produced using this pipeline and different primers. *Created in BioRender*. Kopūstas, (A). (2025) https://BioRender.com/khm2a2s.

Figure 2 illustrates the general procedure (detailed procedure in File S1) for the preparation of several different useful linear DNA fragments including:

1. The λ DNA was digested using Bsp120I restriction endonuclease to reduce concatenation. Follow the Bsp120I vendor's protocol for digestion and deactivation.
2. The 15 kb‐long PCR fragments:
  - digoxigenin–λ DNA 15 kb–biotin produced by a PCR reaction with ol2‐biotin and ol1‐digoxigenin as primers and full‐length λ DNA as a template. Use for either single‐ or double‐end tethering.
  - λ DNA 15 kb–biotin produced by a PCR with ol2‐biotin and ol1 as primers and full‐length λ DNA as a template. Use for single‐end tethering on traptavidin‐coated surface and control of non‐specific interaction with antidigoxin antibody.
  - digoxigenin–λ DNA 15 kb produced by a PCR with ol2 and ol1‐digoxigenin as primers and full‐length λ DNA as a template. Use for single‐end tethering on antidigoxin‐coated surfaces and control of non‐specific interactions with traptavidin.
  - simple λ DNA 15 kb without functional groups produced by a PCR reaction with ol2 and ol1 as primers and full‐length λ DNA as a template. Use for non‐specific binding controls.
3. The 5 kb‐long linear DNA fragments (phiX DNA):
  - phiX DNA–biotin produced by a PCR reaction with ol3‐biotin and ol4 as primers and full‐length phiX DNA as a template. Use for either single‐end tethering on traptavidin‐coated surfaces or control of non‐specific interaction with antidigoxin antibody.
  - Simple phiX DNA without functional groups was produced by a PCR reaction with ol3 and ol4 as primers and full‐length phiX DNA as a template. Use for non‐specific binding controls.

Figure 2 illustrates the general procedure for purification of the DNA fragment of interest after the PCR:

1. Perform agarose gel electrophoresis.
2. Identify the target fragment using a DNA size ruler and cut out the target fragment‐containing band of the gel.
3. Perform gel‐extraction using a DNA gel‐extraction kit.
4. Aliquot the purified target fragment into smaller volumes and keep at −20 °C.

Two ways of preparation of λ DNA for DNA flow‐stretch assays were established: (1) anneal and ligate biotin‐ and digoxigenin‐DNA oligos to the λ DNA cos sites, and purify using chromatography [34, 54], (2) surface‐immobilise biotin‐DNA oligonucleotide (complementary to a cos site), and let λ DNA anneal to this tether in the flowcell [55]. A commercial λ DNA comes as a rather low‐concentration solution (~10 nm) containing a mixture of various‐length fragments. At such low concentrations, loss of DNA to test tube surfaces and pipette tips manifests as a lower surface density of immobilised DNA and an unreproducible result. Additional purification steps further lower the initial concentration. Therefore, we suggest PCR‐amplification of a portion of λ DNA length to >>10 nm, and proper purification using gel‐extraction.

### Surface preparation and flowcell assembly

**Fig. 3:**
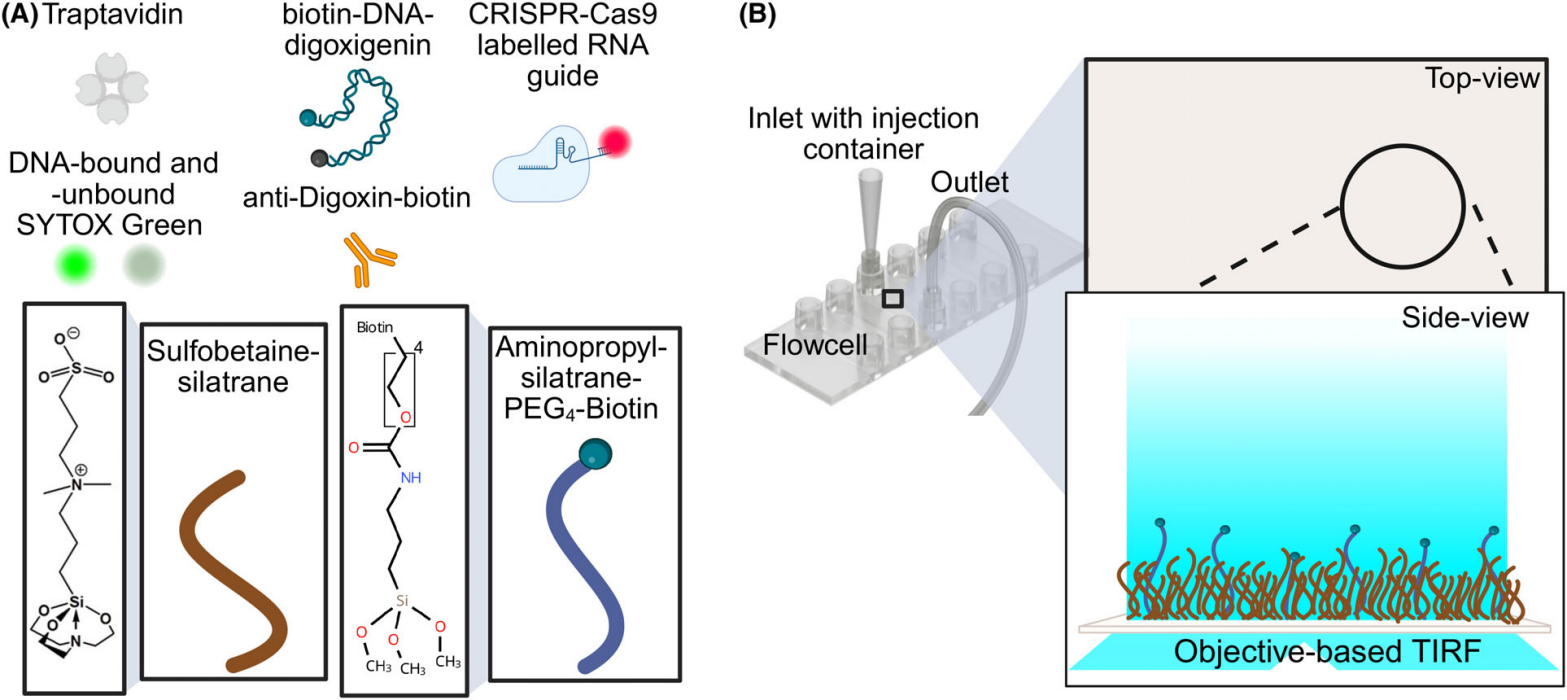
Schematic illustrations of: (A) reagents used to assemble DNA flow‐stretch assay. (B) Flowcell and zoomed views (side‐ and top‐view) of surface with mixed layer composed of sulfobetaine‐silatrane and aminopropyl‐silatrane, to which PEG_4_‐Biotin was conjugated (layer with terminal sulfobetaine and biotin‐PEG_4_ groups) during glass surface modification procedure. *Created in BioRender*. Kopūstas, (A). (2025) https://BioRender.com/a4e0ix6.

**Fig. 4:**
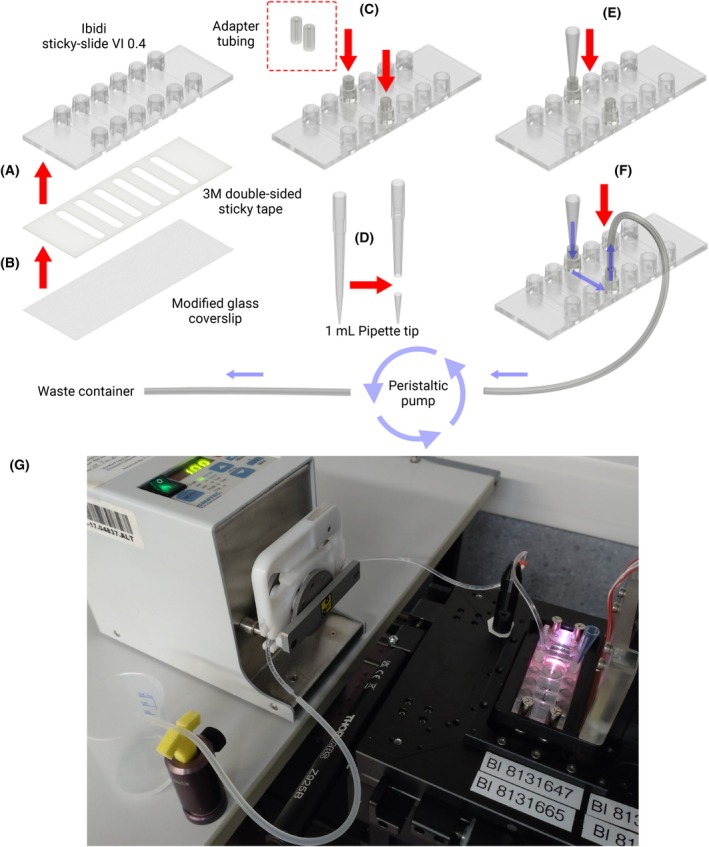
Schematics and photograph illustrating flowcell assembling and interfacing with the pump and microscope. (A, B) Placing double‐sided sticky tape with holes aligning to flowcell channels. (C) Inserting adapter tubings into the inlet and outlet of the flowcell. (D) Cutting a 1 mL pipette tip. (E) Inserting the cut pipette tip (injection reservoir) into the inlet adapter tubing of the flowcell. (F) Connecting the outlet adapter tubing with the tubing of the peristaltic pump. In this solution, the peristaltic pump functions in the suction mode. (G) Photograph showing the flowcell placed on the microscope sample stage and connected to the pump. *Created in BioRender*. Kopūstas, (A). (2025) https://BioRender.com/9jairhk.

The procedures for glass coverslip surface modification (Fig. 3) and flowcell assembly (Fig. 4) are detailed in the File S2 (video with captions).

Typical glass surface modification for the DNA flow‐stretch assay is the so‐called two‐step procedure: (1) silanisation and (2) PEGylation [56, 57, 58]. A common problem with PEG coatings is their degradation over time and issues with reproducibility [59, 60]. Alternatively, a clean glass surface is often simply coated with a BSA/BSA‐biotin layer [61]. This approach suffers from a low level of passivation against nonspecific interactions. Therefore, we switched to newly developed zwitterionic coatings based on sulfobetaine‐silatrane [50] and introduced functional groups for biotin‐DNA immobilisation on traptavidin using aminopropyl‐silatrane conjugated to PEG_4_‐biotin [52, 62].

We prefer to use a commercially available flowcell and reuse it by removing the old tape and attaching new double‐sided sticky tape for DNA flow‐stretch assays. This approach enables us to utilise multiple channels on the same flowcell, allowing for efficient reagent use.

### Single‐tethered DNA flow‐stretch assays

I. Assay with 5‐kb‐long phiX DNA

**Fig. 5:**
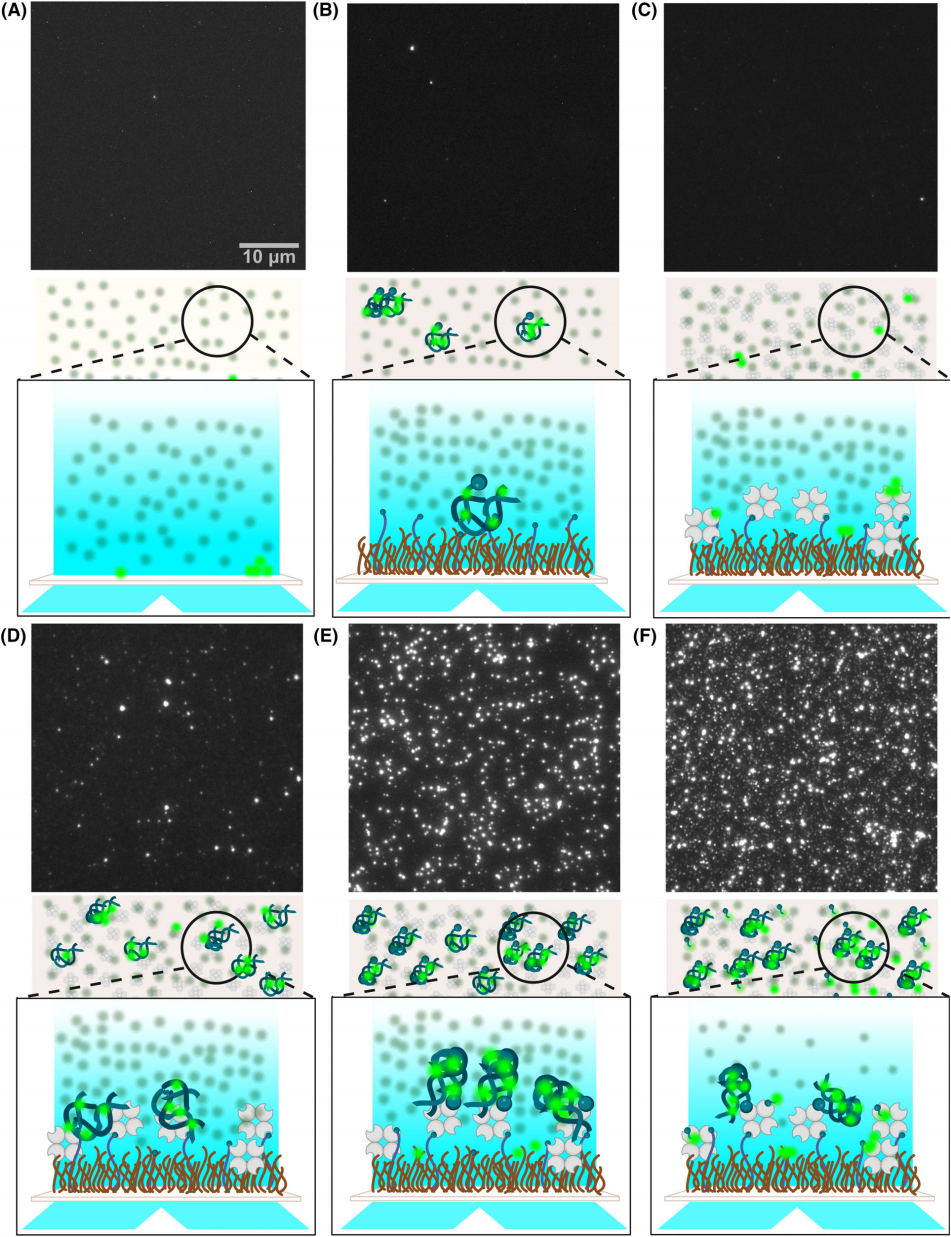
TIRF microscopy images and schematic illustrations of surface modifications, non‐specific SYTOX Green Nucleic Acid Stain binding to surface or proteins, and both specific and non‐specific binding of 5‐kb‐long phiX DNA fragments. (A) SYTOX Green on a clean glass surface. (B) phiX DNA‐biotin on glass surface containing sulfobetaine and biotin‐PEG_4_ terminal groups in the absence of traptavidin. (C) SYTOX Green on the modified glass surface in the presence of traptavidin. (D) phiX DNA (no biotin) on a modified glass surface in the presence of traptavidin. (E) phiX DNA‐biotin on the modified surface in the presence of traptavidin. (F) phiX DNA‐biotin (without gel‐based purification) on traptavidin‐coated modified glass surface. Scale bar 10 μm. SI contains movies with buffer flow showing phiX DNA stretching. See Fig. 3A for the illustration legend. The greyscale of all microscopy images was normalised to the same values. *Created in BioRender*. Kopūstas, (A). (2025) https://BioRender.com/1zvyg3n.

1. → 200 μL buffer1 into the injection container. Avoid introducing air bubbles. If they get introduced, try pipetting them out.
2. Start the pump at a rate of 1 mL·min^−1^. The buffer should fill the entire channel, reach the outlet tubing and still contain approximately 10% of the injection volume. Reverse the flow and give a short pulse backwards into the injection container to remove bubbles that are usually trapped in the adapter tubing. Then, remove it using a pipette. Never dry out the flow cell channel, as this can irreparably damage the surface.
3. Test the nonspecific binding of SYTOX Green:
  - → 300 μL 10 nm SYTOX Green in buffer1. The expected result is illustrated in Fig. 5A.
  - → 200 μL of buffer1 three times.
4. Test phiX DNA‐biotin nonspecific binding to the surface in the absence of traptavidin:
  - → 100 μL ~30 pm phiX DNA‐biotin and incubate > 5 min.
  - → 200 μL buffer1 three times.
  - → 300 μL 10 nm SYTOX Green in buffer1. The expected result is illustrated in Fig. 5B (movie in File S3).
  - → 200 μL buffer1 three times.
5. → 100 μL 0.02 mg·mL^−1^ traptavidin in buffer1.
6. Incubate 10 min.
7. → 200 μL of buffer1 three times.
8. Test nonspecific binding of SYTOX Green to traptavidin:
  - → 300 μL 10 nm SYTOX Green in buffer1. The expected result is illustrated in Fig. 5C.
  - → 200 μL buffer1 three times.
9. Test nonspecific binding of non‐functionalised phiX DNA:
  - → 100 μL ~30 pm phiX DNA without biotin and incubate > 5 min.
  - → 200 μL buffer1 three times.
  - → 300 μL 10 nm SYTOX Green in buffer1. The expected result is illustrated in Fig. 5D (movie in File S4).
  - → 200 μL buffer1 three times.
10. Immobilisation of phiX DNA‐biotin:
  - → 100 μL ~30 pm phiX DNA‐biotin and incubate > 5 min.
  - → 200 μL buffer1 three times.
  - → 300 μL 10 nm SYTOX Green in buffer1. The expected result is illustrated in Fig. 5E (movie in File S5).
  - → 200 μL of buffer1 three times.
11. → buffer that is required for the studied protein's activity.
12. → protein at < 10 nm.
13. → flow‐stretch the DNA and monitor which DNA‐bound protein responds to the flow.

Under buffer flow, our surface‐immobilised DNA molecules stretch efficiently, resulting in short linear DNA fragments that are visible by TIRF microscopy (File S5). A high percentage of flow‐responding DNA fragments is a good indication of both proper surface and DNA preparation. The phiX DNA‐biotin shows very little binding to the modified surface in the absence of traptavidin (Fig. 5B), indicating that our surface modification does not compromise DNA tethering. However, when this surface is covered with traptavidin (Fig. 5D), even nonbiotinylated phiX DNA shows detectable binding. Thus, DNA interacts slightly with such protein‐coated surfaces. Our approach shows very minute nonspecific binding of SYTOX Green in the absence of traptavidin (Fig. 5A). This binding is slightly increased with traptavidin (Fig. 5C), suggesting that these dyes interact with proteins. The PreciseGreen double‐stranded DNA Quantification Reagent shows less interaction with proteins, but compared to SYTOX Green, it is less tested for DNA flow‐stretch assays [63].

II. Assay with full‐length λ DNA

**Fig. 6:**
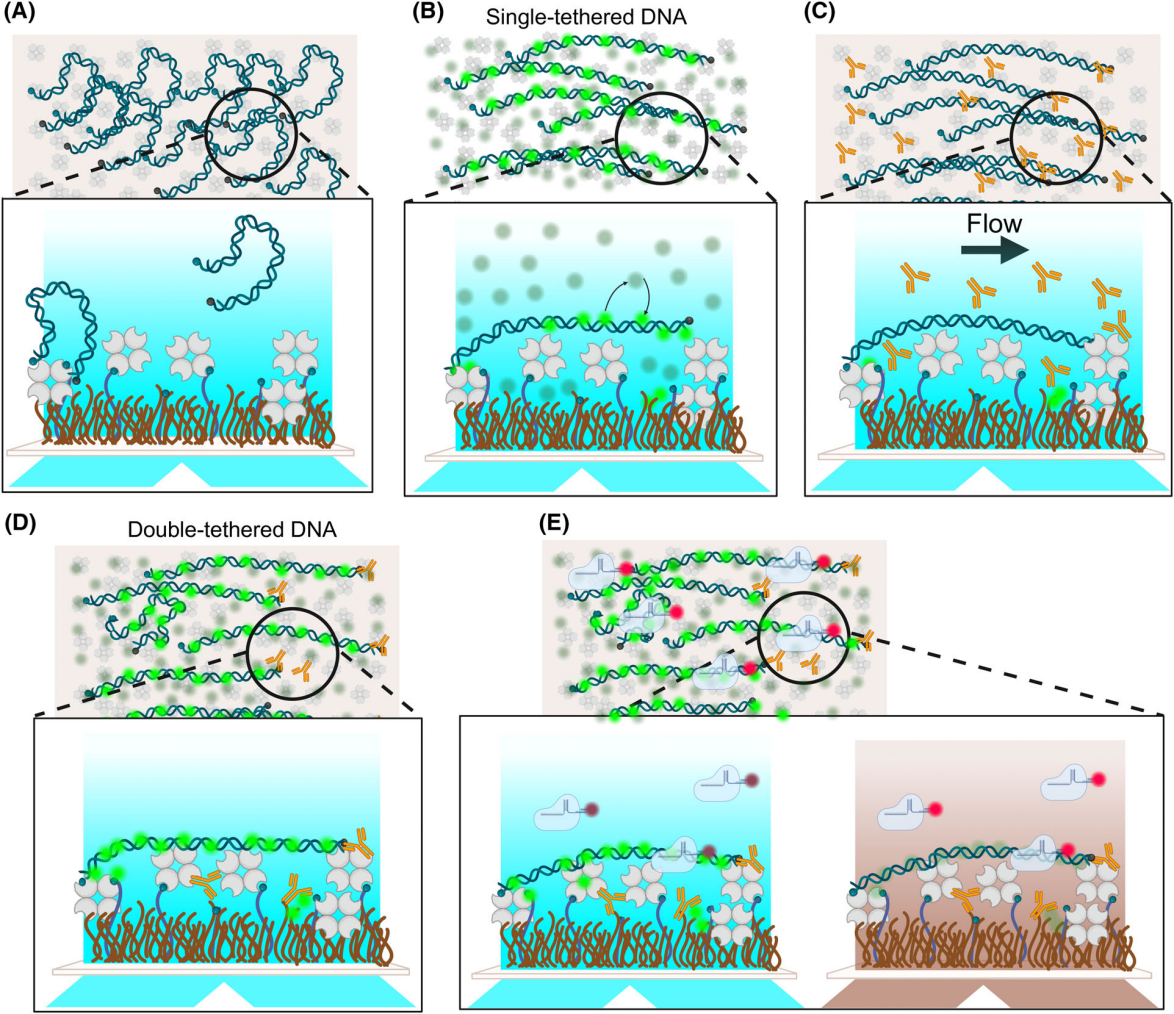
Experiment layout of λ DNA flow‐stretch assay. Schematic illustrations showing: (A) immobilisation step of digoxigenin‐λ DNA‐biotin without flow on traptavidin‐coated surface with terminal sufobetaine and biotin‐PEG_4_ groups. (B) Surface with biotinylated‐end tethered digoxigenin‐λ DNA‐biotin fragments stretched using buffer flow. SYTOX Green is present in the imaging buffer. It intercalates into DNA and becomes brightly fluorescent, while it is strongly quenched in a buffer. Note, some of SYTOX Green interacts with traptavidin and with the surface non‐specifically, and becomes fluorescent too. Only a fraction of injected DNA molecules successfully binds to surface‐immobilised traptavidin, and others are washed away by the buffer flow. (C) Second‐end of λ DNA immobilisation using biotinylated antidigoxin antibody in the presence of continuous buffer flow. Here an excess of antidigoxin‐biotin is injected, and only a small fraction of that successfully binds to both traptavidin and digoxigenated‐end of DNA. There is a fraction of antidigoxin–biotin that remains bound only to traptavidin or to the digoxigenated‐end of DNA. Also, a low fraction of the antibody interacts with the surface nonspecifically. The nonimmobilised antibody fraction is removed by washing the surface with the flow of a buffer. (D) Surface with double‐tethered λ DNA fragments without and with flow look the similar. (E) Surface under either 488 or 638 nm wavelength excitation during fluorescently labelled protein incubation. *Created in BioRender*. Kopūstas, (A). (2025) https://BioRender.com/ju0uhi0.

**Fig. 7:**
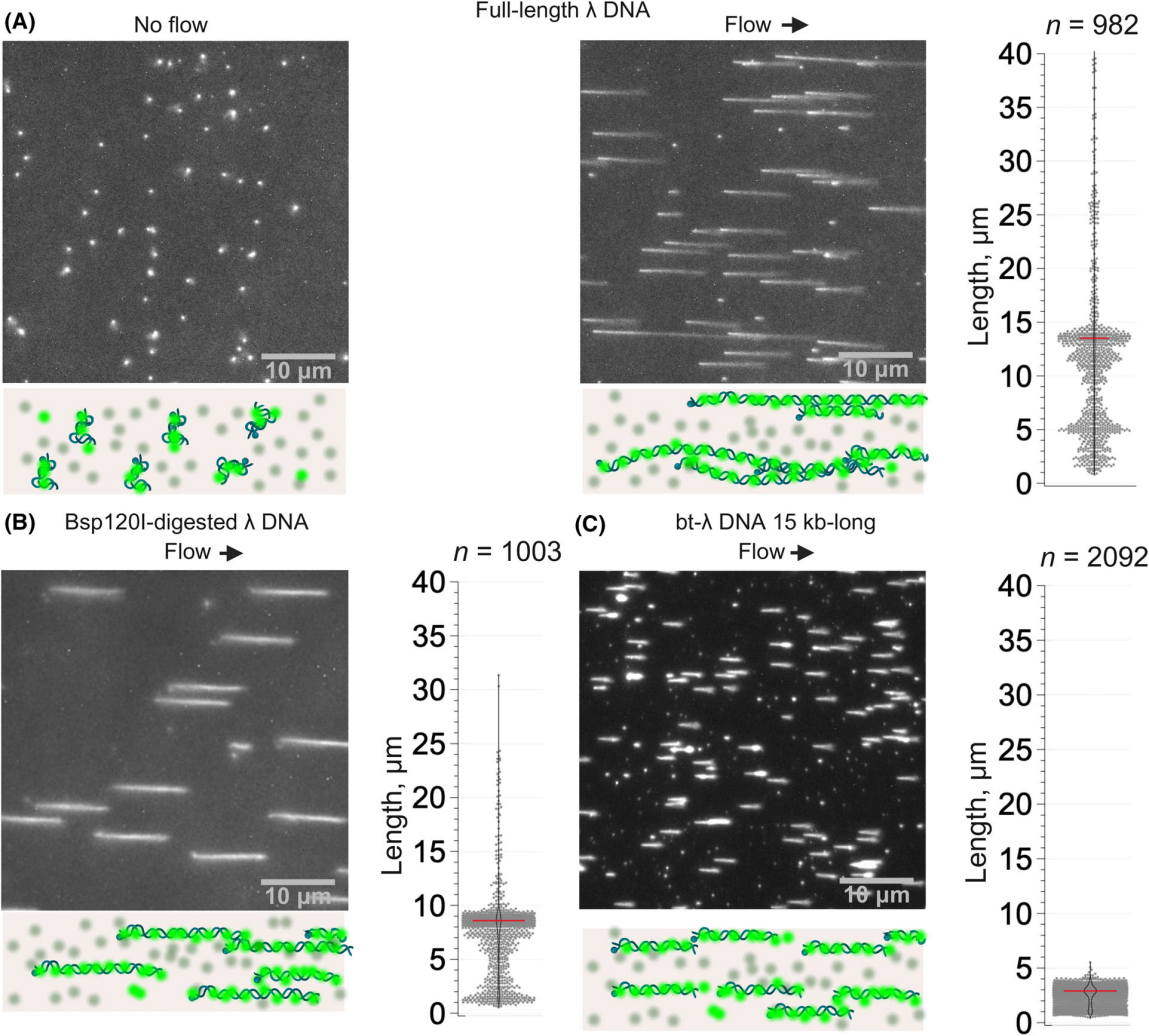
TIRF microscopy images showing cases of λ DNA fragments. (A) Full‐length λ DNA immobilised on biotin‐oligo (complementary to the cos site of λ DNA) via traptavidin on surface containing sulfobetaine and biotin terminal groups. The left panel is without buffer flow, and right ‐ with buffer flow. This illustrates the concatenation of the DNA substrate and length heterogeneity. (B) Treatment of the full‐length λ DNA with restriction endonuclease Bsp120I (unique cleavage site on λ DNA) allows for achieving a more homogeneous DNA length. (C) A PCR produced biotinylated (bt) 15‐kb‐long linear λ DNA fragment immobilised on the surface and stretched with buffer flow. Graphs on the right‐hand side of each panel show the measured DNA length distributions, and the red horizontal line shows the mean target fragment length obtained by fitting the main peak in a 1D histogram of length distribution. The total number (*n*) of analysed individual DNA molecules per each tested condition is indicated above the graphs. Overlapping or aggregated DNA molecules, as well as those that originated from outside the field of view or exhibited double‐end tethering to the surface were excluded from the data analysis. The greyscale of all microscopy images was normalised to the same values. *Created in BioRender*. Kopūstas, (A). (2026) https://BioRender.com/mfkzot3.

1. Perform steps 1–7 from the ‘I. Assay with 5 kb‐long phiX DNA’ protocol.
2. → 100 uL 10 nm ol2‐biotin in buffer1 and incubate > 5 min.
3. → 200 μL buffer1 three times.
4. → 100 μL 30 pm full‐length λ DNA in buffer1 and incubate > 10 min (Fig. 6A).
5. → 200 μL buffer1 three times.
6. → 300 μL 10 nm SYTOX Green in buffer1 (Fig. 6B, Fig. 7A, and movie in File S6).

Typical results show efficient DNA flow‐stretching and various‐length linear DNA fragments (Fig. 7A). DNA extension lengths vary because full‐length λ DNA can concatenate and also contain broken fragments. This complicates studies of DNA‐interacting proteins because the surface‐tethered end of DNA is defined, but the surface‐distant end is not clearly defined.

III. Assay with Bsp120I‐digested λ DNA

1. Perform Steps 1–3 from the ‘II. Assay with full‐length λ DNA’ protocol.
2. → 100 μL 30 pm Bsp120I‐digested λ DNA in buffer1 and incubate > 5 min.
3. → 200 μL buffer1 three times.
4. → 300 μL 10 nm SYTOX Green in buffer1.

Typical results show efficient DNA flow‐stretching and uniform length linear DNA fragments (Fig. 7B, and movie in File S7). Bsp120I has a single recognition site in the λ DNA genome (GGGCCC; bp 10086‐10 091 from the 5′ end), yielding two fragments of linear DNA molecules. Upon surface tethering of the longer fragment (~38500 bp‐long) and flow stretching it, we observe fragments with a uniform extension of ~8–9 μm, compared with heterogeneous lengths of ~13–14 μm observed for nondigested full‐length λ DNA fragments (~48 500 bp) (Fig. 7). However, it is challenging to achieve a high density of surface‐immobilised DNA fragments because the DNA stock concentration is limited. Here, both the surface tethering end of DNA and the surface‐distant ends are more clearly defined.

IV. Assay with 15‐kb‐long λ DNA

1. Perform Steps 1–7 from the ‘Assay with 5 kb‐long phiX DNA’ protocol.
2. Non‐specific and specific immobilisation:
  - For nonspecific binding testing, → 100 μL 30 pm non‐functionalised λ DNA 15 kb or λ DNA 15 kb‐digoxigenin in buffer1.
  - For specific immobilisation, → 100 μL 30 pm biotin‐λ DNA 15 kb in buffer1.
3. Incubate 1 min.
4. → 200 μL buffer1 three times.
5. → 300 μL 10 nm SYTOX Green in buffer1.

Typical results show efficient DNA flow‐stretching and uniform length linear DNA fragments (Fig. 7C and movie in File S8). We achieve a high density of surface‐immobilised DNA fragments. The advantage, compared to the full‐length λ DNA, is the availability of proper controls and the possibility of having both ends clearly defined and functionalised with various moieties. For instance, one can prepare a 15‐kb‐long λ DNA without biotin groups by changing PCR primer ol2‐biotin to ol2, and then test nonspecific binding of such fragment by injecting it at Step 2 of this protocol.

### Double‐tethered 15‐kb‐long λ DNA flow‐stretch assay

1. Perform Steps 1–7 from the ‘I. Assay with 5 kb‐long phiX DNA’ protocol.
2. Nonspecific binding and specific immobilisation:
  - For a nonspecific binding test, → 100 μL 30 pm λ DNA 15 kb‐digoxigenin in buffer1.
  - For specific immobilisation, → 100 μL 30 pm biotin‐λ DNA‐digoxigenin 15 kb in buffer1.
3. Incubate 15 min.
4. → 200 μL buffer1 three times.
5. → 300 μL 10 nm SYTOX Green in buffer1.
6. → 200 μL buffer1 three times.
7. For double‐tethering, inject 1 mL biotinylated antidigoxin antibody into the injection container and apply a buffer flow of approximately > 1 mL·min^−1^ to extend DNA molecules, allowing the antibody to bind to traptavidin and interact with the digoxigenin‐labelled end of the DNA. Fill the injection container with buffer1 until at least 10 mL of buffer has flowed through. Then step on the pump (Fig. 6 C,D).
8. → 300 μL 10 nm SYTOX Green in buffer1.

Typical results show stretched DNAs with uniform length that wiggle slightly without the flow (see movie in File S9). Note, it is hard to achieve 100% of double tethering, and even 50% of double‐tethered DNAs (of all surface‐immobilised DNAs) is a good result. A limiting factor is free antidigoxin–biotin competing for surface‐immobilised traptavidin sites, which can reduce DNA double‐tethering yield. Accordingly, flow rate optimisation is important: high flow confines the polymer fluctuation and reduces end–surface encounters needed for second‐end capture, whereas very low flow can lead to insufficient and heterogeneous DNA extension (variable apparent lengths), often compromising visualisation of DNA‐interacting proteins under TIRF.

### Revealing the interactions of *d*Cas9 with DNA by DNA flow‐stretch assays

**Fig. 8:**
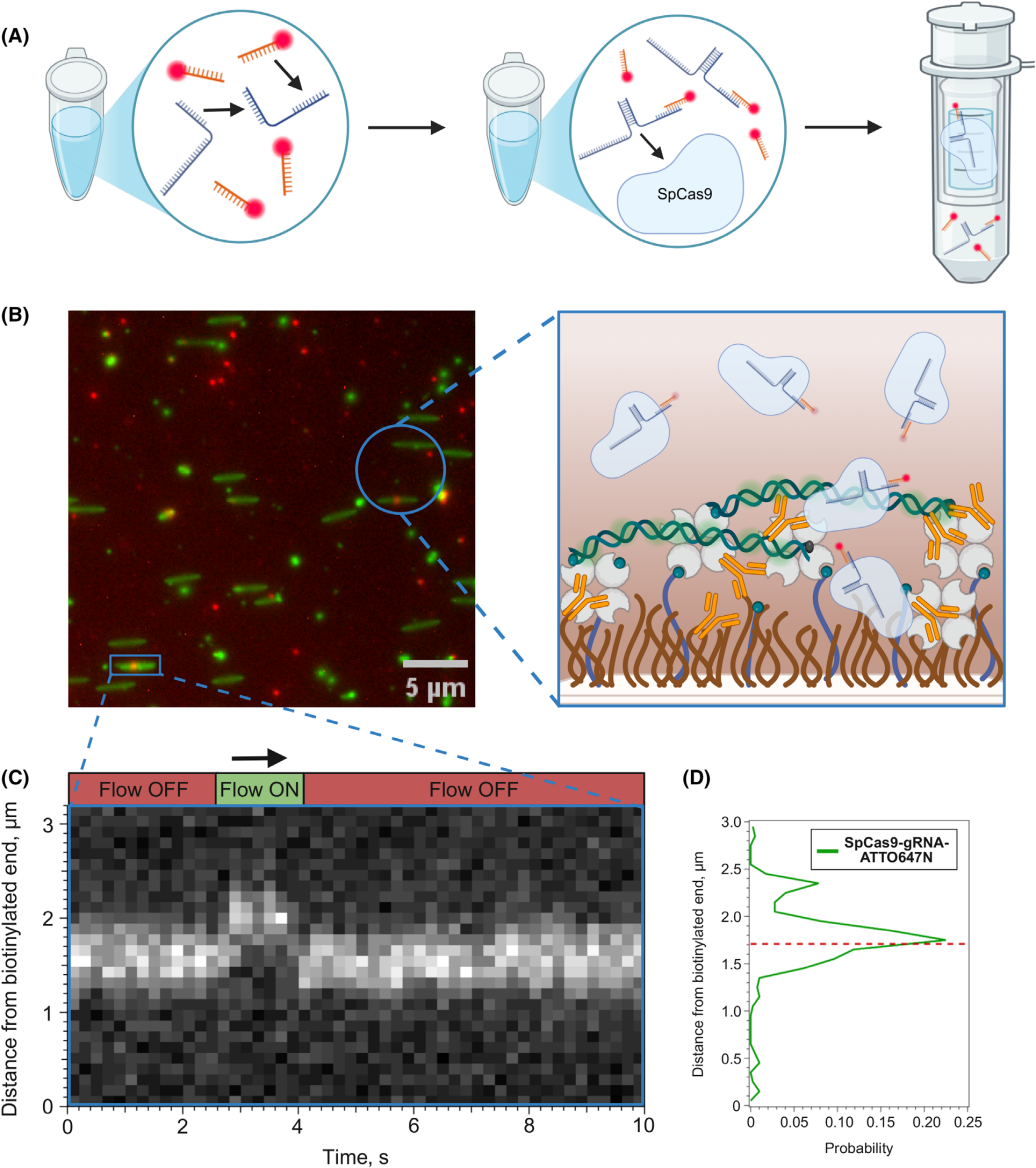
Exploring catalytically inactive dCas9‐DNA interactions via single‐molecule DNA flow‐stretch assays. (A) Optimised workflow of preparing dCas9 protein for imaging its interaction dynamics with DNA at the single‐molecule level: annealing and fluorescent labelling of guide RNA (left), loading of dCas9 with the labelled gRNA (centre), and ultrafiltration‐based purification of the assembled labelled dCas9 RNP complex (right). (B) Merged dual‐colour TIRF microscopy image (left) of a typical single‐molecule double‐tethered DNA flow‐stretch assay that has been employed for studying a fluorescently labelled DNA‐interacting protein of interest. Here, SYTOX Green‐stained digoxigenin‐λ DNA 15 kb‐biotin (green colour) and dead dCas9‐gRNA‐ATTO647N complex (red colour) was used. In this case, two‐channel images were acquired without the buffer flow. Schematic illustration (right) of the selected region of interest (blue circle) indicates several possible fates characteristic to the observed biomolecules. (C) Representative kymograph of individual dead dCas9 protein bound to its target site present in the 15 kb‐long λ DNA fragment. Double‐tethered DNA molecule, which was specifically bound by dead dCas9, used for kymograph building is marked with a blue rectangle in B) panel. (D) Histogram of the experimentally measured ATTO647N‐labelled dead dCas9 RNP complex's binding positions on the surface‐immobilised digoxigenin‐λ DNA 15 kb‐biotin molecules. In this design, the protein's on‐target binding site (red dashed line) is located at a distance of 8.8 kb from the biotinylated end of such 15 kb‐long λ DNA fragment. *Created in BioRender*. Kopūstas, (A). (2025) https://BioRender.com/idhzkud.

Figure 8A illustrates the principal steps of fluorescently preparing dCas9 RNP complex for SM‐level studies employing DNA flow‐stretch assays:

1. Labelling gRNA of dCas9 (in a conical screw cap tube):
  - 5 μm crRNA,
  - 5 μm tracrRNA containing an extension at 3′ end,
  - 5 μm ATTO647N‐labelled DNA oligo,
  - 1X annealing buffer,
  - DEPC‐treated water.
2. Boil 300 mL of distilled water in any standard electric kettle, let it rest for a few seconds and then pour the hot water into a glass beaker.
3. Insert the tube into a floating foam tube rack and place it in the beaker with hot water.
4. Leave it at room temperature for at least 3 h to cool down the water to room temperature.
5. Assembling dCas9 RNP complex (in a 0.2 mL PCR tube consisting of):
  - 100 nm dCas9 protein;
  - 700 nm ATTO647N‐labelled gRNA;
  - 1X assembly buffer;
  - 1 mm DTT;
  - DEPC‐treated water.
6. Incubate at 37 °C > 30 min.
7. Mix 20 μL of the dCas9 RNP complex with 430 μL of 1X assembly buffer supplemented with 1 mm DTT.
8. Purify the prepared sample using a centrifugal concentrator: 14 000 rcf, 12 min, RT. By spinning 450 μL of the initial sample volume, the typical retrieved volume of the final concentrate is ~17.5 μL.

The rationale behind choosing to anneal crRNA and tracrRNA (instead of using a single‐guide RNA) lies in our aim to test different targets in a cost‐effective manner. In this case, one only needs to order shorter crRNAs, while tracrRNA is the same. Also, this is an architecture of a guide RNA that is found in a natural CRISPR‐Cas9 system.

At this point, there are two main options of using this RNP complex for DNA flow‐stretch assays: (1) inject it into the flowcell containing already prepared double‐tethered 15‐kb‐long λ DNAs (described in the previous subsection). This option is perfect for observing and measuring the dynamic nature of dCas9 interactions with DNA, or (2) robustly visualise dCas9 binding to individual surface‐tethered λ DNA 15‐kb molecules by these steps:

1. Load the purified fluorescently‐labelled dCas9 RNP complex onto the functionalised λ DNA 15‐kb fragment (in a test tube, 100 μL mixture):
  - 10 pm digoxigenin‐λ DNA 15 kb‐biotin;
  - 1 nm dCas9‐gRNA‐ATTO647N;
  - 1X reaction buffer;
  - 1 mm DTT;
  - Ultrapure water.
2. Incubate the reaction mixture at 37 °C > 30 min.
3. Perform Steps 1–7 from ‘I. Assay with 5 kb‐long phiX DNA’.
4. → 100 μL 1X reaction buffer supplemented with 1 mm DTT.
5. → 100 μL prepared reaction mixture (from step 2.) supplemented with 30 nm SYTOX Green and incubate > 10 min.
6. To perform the flow stretching of single‐tethered DNA molecules, → 300 μL 1X reaction buffer supplemented with 1 mm DTT and 30 nm SYTOX Green.
7. To immobilise the second‐end of DNA on the surface, refer to steps 6–8. of ‘Double‐tethered 15 kb‐long λ DNA flow‐stretch assay’ subsection.

There are three crucial aspects to take notice of when trying to distinguish between the DNA‐bound and nonspecifically surface‐adsorbed states of the fluorescently labelled dCas9 RNP complex. The first clue for assigning dCas9 target‐bound spots is whether the measured position along the DNA substrate matches the expected binding position of the protein, which can be benchmarked in Fig. 8C. Second, follows the responsiveness of the dCas9 RNP complex's fluorescent spot to the buffer flow. If such a spot moves back and forth in accordance with the user‐chosen buffer flow pulses, as the surface‐tethered DNA molecules typically do, then it is highly likely that the observed spot represents dCas9 bound to DNA. Otherwise, the motionless spots usually correspond to non‐specific binding of the protein to the surface. The third hint is the visual quality of the fluorescent spot shape characteristic of the observed spot. If the spot of interest exhibits a sharp and static fluorescent spot with a relatively intense fluorescence signal, such a spot is probably of non‐specific nature. The shape of fluorescent spots that can be reliably attributed to the DNA‐bound dCas9 protein generally appears to be blurry, wobbly and more spread out, together with a lower fluorescence intensity. The latter clue also depends on the exposure time of a camera.

### TIRF microscopy

DNA flow‐stretch assays, if only labelled DNA needs to be measured, require standard single‐molecule fluorescence microscopes. However, if a labelled protein needs to be measured, a standard objective‐based TIRF set‐up is required. For imaging, perform these steps:

1. Set up a TIRF microscope to ensure excitation at the critical angle or slightly above it before adding the actual sample. Follow instructions provided by the producer of the microscope or elsewhere published [64, 65, 66]. For these studies, we are utilising our open‐source microscopy solution, miEye [66].
2. Add a drop of suitable immersion oil onto the oil‐immersion objective.
3. Place the flow cell into the sample holder of the microscope (Fig. 4G).
4. Establish contact between the bottom surface of the flowcell and the oil on the objective.
5. Once DNA is stained by SYTOX Green, turn on a 488‐nm wavelength laser and find the Z‐axis position of the surface. This is usually achieved by trying to sharpen out some surface‐bound fluorescent spots.
6. Establish a buffer1 containing SYTOX Green flow to stretch DNA and move ~500 nm higher into the solution to make the stretched DNAs look sharper.
7. If a microscope has *Z*‐axis drift correction, enable it for minimal focus drift during data acquisition.
8. Set camera exposure time to 100 ms, capturing frames at a rate of 10 frames per second.
9. Acquire data and store it in TIFF format for further data analysis.

### Data analysis

For measuring the length of stretched DNA, various manual or semi‐automated tools are available. One can use FiJi/ImageJ for manual DNA length measurement using a line tool, and then draw line profiles to extract the length of DNA [67, 68]. The same procedure can be performed using automated tools that exist as Fiji plug‐ins, such as the DNA Finder tool, which is present in the Mars plugin [69]. There are also other algorithms and AI‐based tools dedicated to very similar purposes, such as DNA fibre analysis in the single‐molecule fluorescence microscopy data [70, 71, 72]. Based on our experience, the precision of DNA length measurements generally depends on factors such as DNA molecule length (the shorter it is, the more precise its measurement), type of DNA immobilisation (compared to the double‐tethered DNA, single‐tethered flow‐stretched DNA molecules tend to show blurriness on their free end in the averaged TIRF microscopy images, which introduces extra uncertainty in the measurement) and the contrast (signal‐to‐noise ratio) of fluorescently labelled individual DNA molecules (this is particularly important for high‐throughput semi‐automated analysis since the aforementioned tools tend to extract less accurate results when given low contrast signal‐to‐noise ratio datasets). For the DIP binding position, binding event durations, and the speed of translocation/sliding, automated software that utilises 2D Gaussian fitting and single‐molecule tracking must be employed. Most laboratories use custom‐written data analysis code for this purpose, and some are available as open‐source solutions [73, 74].

## Tips & Tricks/troubleshooting

1. It is crucial to gel‐purify DNA fragments to avoid fluorescence background from other DNA products and primers that may be present in the DNA preparation (Fig. 5F and movie in File S10). DNA intercalating dyes (e.g., Sytox Green) can bind to various nonspecific double‐stranded DNA fragments, which may be amplified during PCR, and also to single‐stranded DNA primers to some extent. Because in a PCR mixture usually there is an excess of ssDNA primers compared to dsDNA fragments of interest, the primers give detectable background. In our experience, the standard PCR mix purification kits or spin columns leave a trace amount of ssDNA primers in the final purification product, and since the downstream applications and methods in use here are capable of single‐molecule detection, the remaining rather small quantities of primers are still typically visible as an overall fluorescence background during imaging.
2. If the assay does not achieve a high immobilised DNA density, use fluorescently labelled traptavidin and check if it binds to the modified glass surface. Adjust the traptavidin incubation time and concentration to control the tether density and prevent clustering.
3. If the nonspecific binding of DNA is high, try different surface passivation methods (e.g., adding Tween‐20) [61], use a fresh traptavidin aliquot, and fresh buffers.
4. If the nonspecific binding of a DNA‐interacting protein is too high. Check whether the protein does not have an avi‐ or strep‐tag (if yes, it will bind to traptavidin on the surface). Use a protein that has already been tested (e.g., fluorescently labelled BSA) to determine how much it binds to your surface; it may be that your DNA‐interacting protein is too sticky. If so, try adding BSA or Tween‐20 into your imaging buffer.
5. If the fluorescence stability of the protein label or DNA staining dye is poor, adjust laser power and exposure time of the camera (or frame rate) to minimise bleaching while maintaining good contrast. If that does not help, try changing the dye.
6. Apparent photo‐cleavage of SYTOX Green‐labelled DNA under prolonged illumination is possible. Try to adjust exposure time of the camera and laser power to reduce this effect. Also, consider using oxygen scavenger systems [63]. Just recently, it was demonstrated that Na2SO3 supplementation can also be helpful in such applications [75].
7. DNA should be stretched to 80% of its theoretical length. So adjust flow rates between 0.5 and 2 mL·min^−1^ to extend DNA molecules properly. The 80% stretched‐DNA represents the maximum extension achievable before the force required to stretch the molecule further increases exponentially. Pushing beyond this limit yields diminishing returns in linearity while drastically increasing the risk of tether rupture or DNA breakage. Also, DNA‐interacting proteins recognise B‐form DNA. If the DNA is hyperstretched (approaching 100% or entering S‐phase), the helical pitch changes, potentially inhibiting protein binding or causing artefacts in the interaction kinetics. Keeping the DNA at 80% ensures it remains in a canonical B‐form helix accessible to biological machinery [60].
8. Quantification of molecular behaviours relies heavily on robust image acquisition and analysis pipelines. Try finding one that works best, and compare the results you get from a hand‐based analysis with those from automated analysis.

## Conflict of interest

The authors declare no conflict of interest.

## Author contributions

AKG, MNA, AK, MT, UB optimized the protocols and performed the experiments. AKG, MT, MNA, AK, VTV wrote the initial draft of the manuscript and prepared and edited the figures. MT conceived and designed the study. MT supervised the project involving the experiments and the corresponding data analysis. All authors contributed to reviewing and editing the manuscript and reviewing the figures.

## Supporting information


**File S1.** Detailed procedure for preparation of phiX and 15 kb λ DNAs.

**File S2.** Video showing glass coverslip surface modification and flowcell assembling procedure.

**File S3.** Gif animated image showing flow‐stretching of biotin‐phiX DNA non‐specifically immobilised on the surface in the absence of traptavidin. Exposure time of a frame 100 ms, and 10 frames per second rate.

**File S4.** Gif animated image showing flow‐stretching of phiX DNA without biotin group non‐specifically immobilised on the surface in the presence of traptavidin. Exposure time of a frame 100 ms, and 10 frames per second rate.

**File S5.** Gif animated image showing flow‐stretching of biotin‐phiX DNA specifically immobilised on the surface. Exposure time of a frame 100 ms, and 10 frames per second rate.

**File S6.** Gif animated image showing flow‐stretching of full‐length λ DNA specifically immobilised on the surface. Exposure time of a frame 100 ms, and 10 frames per second rate.

**File S7.** Gif animated image showing flow‐stretching of Bsp120I‐digested λ DNA specifically immobilised on the surface. Exposure time of a frame 100 ms, and 10 frames per second rate.

**File S8.** Gif animated image showing flow‐stretching of λ DNA 15 kb specifically immobilised on the surface. Exposure time of a frame 100 ms, and 10 frames per second rate.

**File S9.** Gif animated image showing flow‐stretching of double‐tethered biotin‐λ DNA 15 kb‐digoxigenin. Exposure time of a frame 100 ms, and 10 frames per second rate.

**File S10.** Gif animated image showing flow‐stretching of biotin‐phiX DNA, which was not purified using gel‐extraction, immobilised on the surface. Exposure time of a frame 100 ms, and 10 frames per second rate.

## Data Availability

The data that support the findings of this study are available from the corresponding author upon reasonable request.
