# Supplementary material for: Single‐molecule DNA flow‐stretch assays for high‐throughput DNA–protein interaction studies
Source: FEBS Open Bio. 2026 Feb 19:10.1002/2211-5463.70211. Online ahead of print. doi: 10.1002/2211-5463.70211 (PMC13398501; doi:10.1002/2211-5463.70211)
Supplement: Supplementary file 1 — File S1. Detailed procedure for preparation of phiX and 15 kb λ DNAs. [file FEB4-9999-0-s004.pdf]

Preparation of phiX DNA and 15 kb λ DNA

A. phiX PCR — Reaction setup (50 µL total)

Component	Stock conc.	Final conc.	Volume (µL)
Q5 Reaction Buffer	5×	1×	10.0
dNTPs	10 mM each	200 µM each	1.0
Forward primer	10 µM	0.5 µM	2.5
Reverse primer	10 µM	0.5 µM	2.5
Template DNA (phiX RF I)	0.5 µg/µL	~10 ng per rxn	1.0
Q5 High-Fidelity DNA Polymerase	2 U/µL	0.02 U/µL	0.5
Nuclease-free water	—	—	32.5
**Total**	—	—	**50.0**

Cycling conditions (optimized for Q5):

1. Initial denaturation: 98°C — 30 s (1 cycle)
2. Denaturation: 98°C — 10 s
3. Annealing: 70°C — 15 s
4. Extension: 72°C — 2.5 mins
5. Repeat steps 2–4 for 30–35 cycles (use 30 first; increase only if yield is low)
6. Final extension: 72°C — 10 mins (1 cycle)
7. Hold: 4°C — ∞

B. 15 kb λ PCR — Reaction setup (50 µL total)

Component	Stock conc.	Final conc.	Volume (µL)
5× Q5 Reaction Buffer	5×	1×	10.0
10 mM dNTP Mix	10 mM each	200 µM each	1.0
Forward primer	10 µM	0.5 µM	2.5
Reverse primer	10 µM	0.5 µM	2.5
Template DNA (λ)	0.3 µg/µL	~10–50 ng per rxn	~1.0
Q5 High-Fidelity Polymerase	2 U/µL	0.02 U/µL	0.5
Nuclease-free water	—	—	up to 50.0
**Total**	—	—	**50.0**

Cycling conditions (optimized for Q5):

1. Initial denaturation: 98°C — 30 s (1 cycle)
2. Denaturation: 98°C — 10 s
3. Annealing: 64°C — 20 s
4. Extension: 72°C — 8 min
5. Repeat steps 2–4 for 25 -30 cycles
6. Final extension: 72°C — 10 min (1 cycle)
7. Hold: 4°C — ∞

C. Post-PCR purification and gel extraction

1. PCR cleanup (column): After amplification, clean the PCR product using the Thermo Fisher Scientific PCR purification column (follow manufacturer protocol for 50 µL reactions). Elute in the recommended elution volume (e.g., 30–50 µL nuclease-free water or 10 mM Tris pH 8.0).

2. Gel electrophoresis: Prepare 0.8% agarose gel (in 1× TAE or 1× TBE) for size separation of long products. Mix sample with 6× loading dye, load cleaned PCR product or uncleaned PCR (as preferred), and run at an appropriate voltage to separate large fragments (typical run: 30–40 min depending on gel size and voltage). Include a suitable high-molecular-weight ladder

3. Band excision & gel extraction: Excise the band of the expected size and perform gel extraction using Thermo Fisher Scientific Gel Extraction Kit, #K0692, following the manufacturer's instructions. Elute in the kit-recommended volume .

4. Concentration measurement: Measure DNA concentration using Nanodrop and/or Qubit. Record both if available; Qubit is preferred for accurate concentration of dsDNA.

Expected final concentrations (from our preparations):

- phiX product: ~4–6 ng/µL after gel extraction and elution.
- 15 kb λ product: ~30–50 ng/µL after gel extraction and elution.

D. Quality control (QC)

- Visualize purified products on agarose gel (0.8% for large fragments; 1% for smaller fragments) to confirm single, appropriately sized bands and absence of major smear or non-specific bands.
- Use Qubit dsDNA HS assay for accurate concentration if available; Nanodrop readings can be inflated by salts/contaminants.
- If desired, perform densitometric quantification (e.g., Fiji/ImageJ) of gel bands and report approximate ng recovered relative to ladder standards.

E. Storage

- Store purified DNA in –20°C for short-term or –80°C for long-term.
- Prepare aliquots to avoid repeated freeze–thaw cycles. Typical aliquot volumes: 5–50 µL depending on downstream needs.

F. Troubleshooting & practical notes (brief)

- Low yield (phiX): check annealing temperature and primer integrity; although a 70°C anneal was optimized here for phiX with the listed primers and Q5, if yield is low try a gradient from 66–72°C or increase cycle number to 35.

- Low yield (15 kb λ): long amplicons are sensitive to template quality and enzyme processivity. Ensure high-quality template, consider running multiple replicate reactions and pooling, and verify extension time (8 min per cycle) — increase extension by 1–2 min if required.

- Gel extraction losses: gel extraction typically reduces concentration — expect recovery losses. If you need higher concentrations, elute in smaller volume (e.g., 30 µL) or concentrate eluate by ethanol precipitation or SpeedVac. Reported final concentrations above reflect post-extraction values.

- Contamination / non-specific bands: run no-template controls and adjust annealing/primer design if non-specific products persist.

- Quantitation discrepancies: prioritize Qubit for low-concentration samples; Nanodrop can overestimate due to contaminants.
