# Supplementary figures and images for: Single‐molecule DNA flow‐stretch assays for high‐throughput DNA–protein interaction studies
Source: FEBS Open Bio. 2026 Feb 19:10.1002/2211-5463.70211. Online ahead of print. doi: 10.1002/2211-5463.70211 (PMC13398501; doi:10.1002/2211-5463.70211)

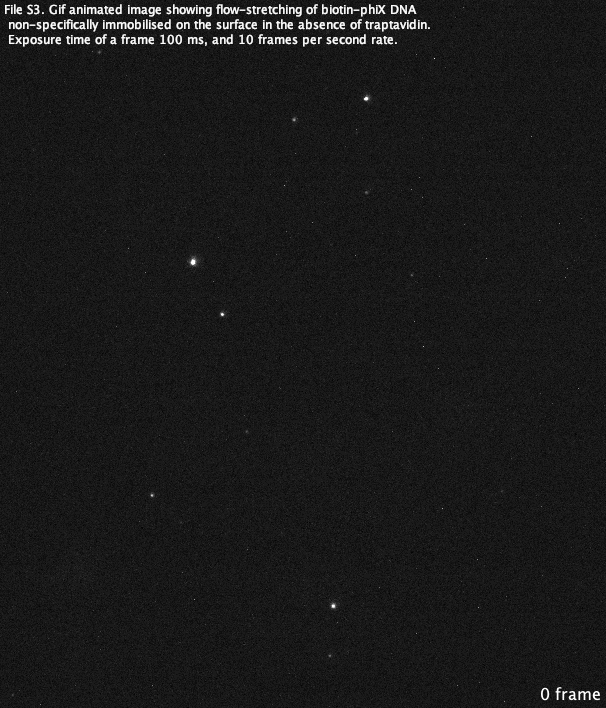

Supplement: Supplementary file 3 — File S3. Gif animated image showing flow‐stretching of biotin‐phiX DNA non‐specifically immobilised on the surface in the absence of traptavidin. Exposure time of a frame 100 ms, and 10 frames per second rate. [file FEB4-9999-0-s005.gif]

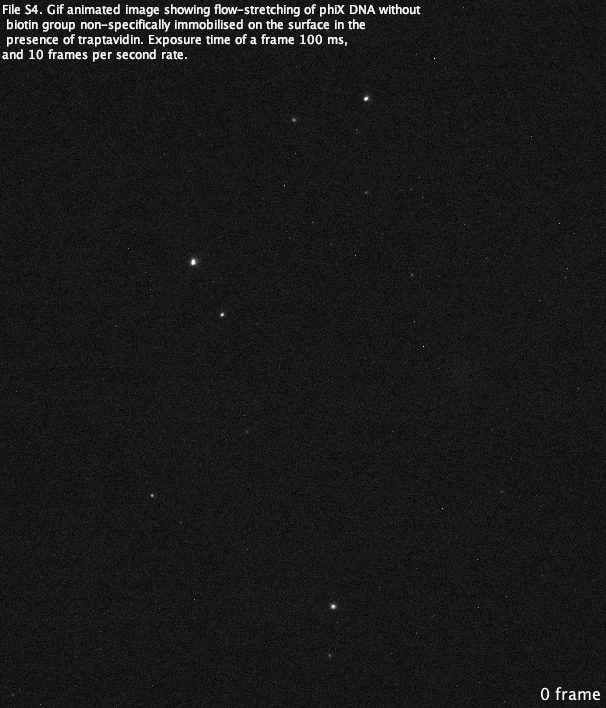

Supplement: Supplementary file 4 — File S4. Gif animated image showing flow‐stretching of phiX DNA without biotin group non‐specifically immobilised on the surface in the presence of traptavidin. Exposure time of a frame 100 ms, and 10 frames per second rate. [file FEB4-9999-0-s009.gif]

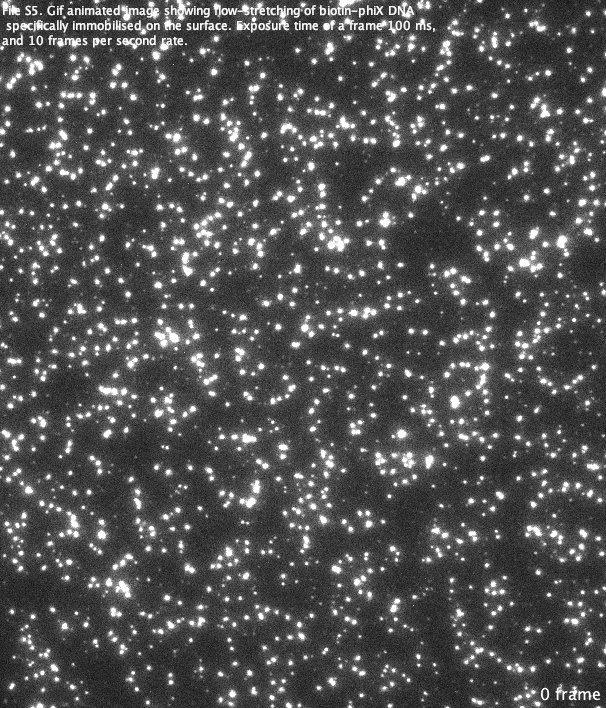

Supplement: Supplementary file 5 — File S5. Gif animated image showing flow‐stretching of biotin‐phiX DNA specifically immobilised on the surface. Exposure time of a frame 100 ms, and 10 frames per second rate. [file FEB4-9999-0-s006.gif]

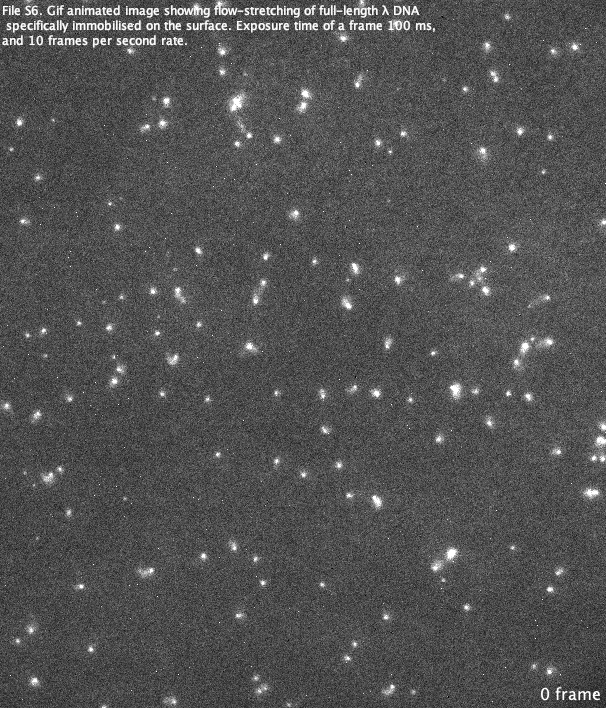

Supplement: Supplementary file 6 — File S6. Gif animated image showing flow‐stretching of full‐length λ DNA specifically immobilised on the surface. Exposure time of a frame 100 ms, and 10 frames per second rate. [file FEB4-9999-0-s007.gif]

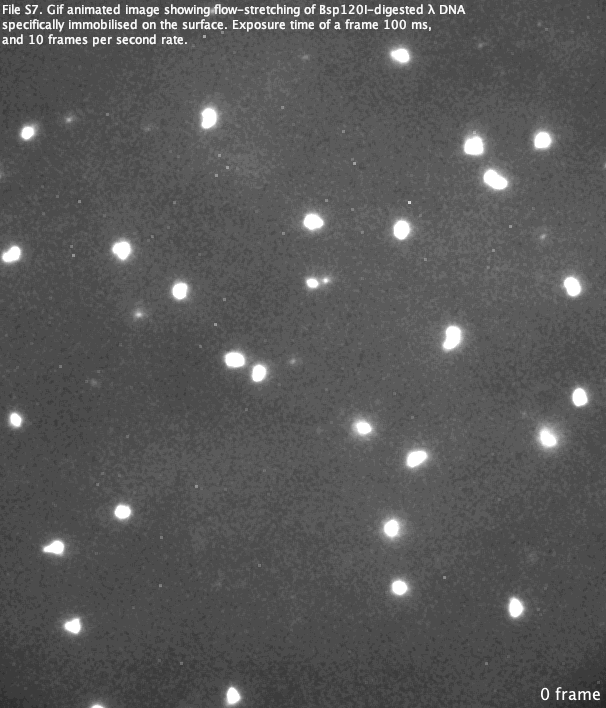

Supplement: Supplementary file 7 — File S7. Gif animated image showing flow‐stretching of Bsp120I‐digested λ DNA specifically immobilised on the surface. Exposure time of a frame 100 ms, and 10 frames per second rate. [file FEB4-9999-0-s003.gif]

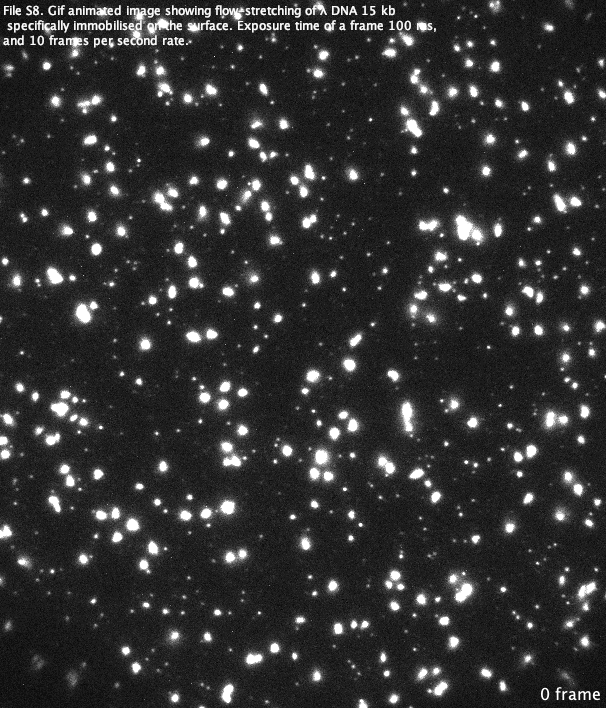

Supplement: Supplementary file 8 — File S8. Gif animated image showing flow‐stretching of λ DNA 15 kb specifically immobilised on the surface. Exposure time of a frame 100 ms, and 10 frames per second rate. [file FEB4-9999-0-s010.gif]

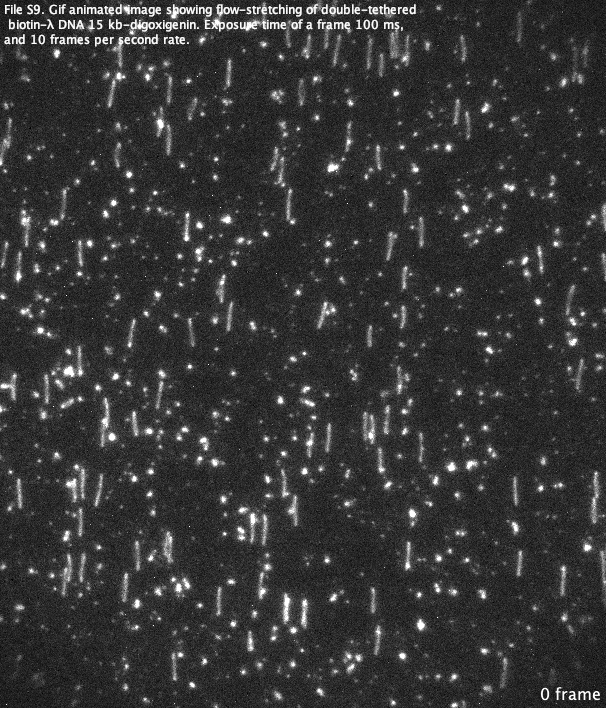

Supplement: Supplementary file 9 — File S9. Gif animated image showing flow‐stretching of double‐tethered biotin‐λ DNA 15 kb‐digoxigenin. Exposure time of a frame 100 ms, and 10 frames per second rate. [file FEB4-9999-0-s002.gif]

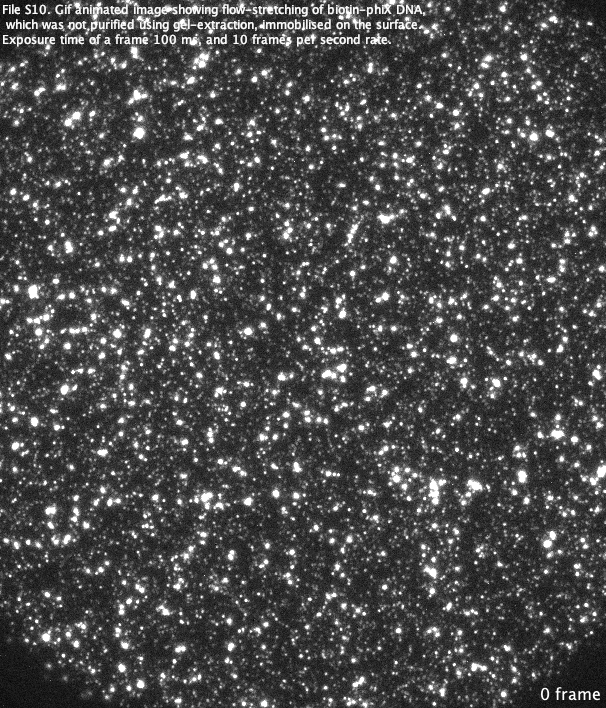

Supplement: Supplementary file 10 — File S10. Gif animated image showing flow‐stretching of biotin‐phiX DNA, which was not purified using gel‐extraction, immobilised on the surface. Exposure time of a frame 100 ms, and 10 frames per second rate. [file FEB4-9999-0-s008.gif]
